# Have You Heard That—“GOSSIP”? Gossip Spreads Rapidly and Influences Broadly

**DOI:** 10.3390/ijerph182413389

**Published:** 2021-12-20

**Authors:** Rezwan Ullah, Muhammad Zada, Imran Saeed, Jawad Khan, Muhammad Shahbaz, Alejandro Vega-Muñoz, Guido Salazar-Sepúlveda

**Affiliations:** 1 School of Management and Economics, Beijing Institute of Technology, Beijing 100081, China; rezwanullah1990@yahoo.com; 2 Postdoc Business School, Henan University, Kaifeng 475000, China; 3 Institute of Business and Management Sciences, The University of Agriculture, Peshawar 25130, Pakistan; imranktk1984@gmail.com; 4 Department of Business Administration, Iqra National University, Peshawar 25100, Pakistan; jawadmarwat1@gmail.com; 5 Public Policy Observatory, Universidad Autónoma de Chile, Santiago 7500912, Chile; alejandro.vega@uautonoma.cl; 6 Departamento de Ingeniería Industrial, Facultad de Ingeniería, Universidad Católica de la Santísima Concepción, Concepción 4090541, Chile; gsalazar@ucsc.cl

**Keywords:** negative workplace gossip, ego depletion, political acts, emotional intelligence, AET

## Abstract

This study examines the impact of negative workplace gossip (NWG) on employee political acts (PA) and the role of ego depletion (ED) as a mediator. We also examined the indirect impact of NWG on PA through ED controlled by emotional intelligence (EI). A three-wave time-lagged study (paper-pencil based) was performed with 277 employees from various private organisations in Islamabad, Pakistan. The current data were gathered in three phases to reduce common method bias. Study results indicate that NWG positively affects employees’ PA. The authors also found ED as a potential mediator in the association between NWG and PA. In addition, the results also indicate the indirect effect of NWG on targets’ PA via ED is reduced by targets’ EI, with the result that this connection is weak when targets’ EI is high. Because this research is limited to a single region of Pakistan, particularly Islamabad, its findings cannot be comprehensive. Future studies should use a larger sample size to accomplish the same study. Future studies may include more organisations (that is, Public) to conduct a comparative analysis of the public and private sectors. This article, based on the affective events theory (AET), argues that EI should be utilised to mitigate the effects of NWG. Along with our significant and relevant theoretical contributions, we provide novel insights into the body of knowledge on how managers may prevent or minimise such PA. The current study results support all direct and indirect hypothesised connections, with important implications for theory and practice. A review of the existing literature indicates that EI may be associated with a reduction in employees’ ED; however, EI has not been used as a moderator in mitigating the influence of NWG, ED, and PA in the past.

## 1. Introduction

GOSSIP, or the telling or repeating of stories about others, is a worldwide social phenomenon that continues to play a significant part in today’s life. According to previous studies, employees spend more than half of their conversation time in gossiping [1]. According to researchers, 14% of office coffee-break conversations are gossip, while about 66% of general conversion between employees involves social themes, including gossip about other co-employees social lives [2]. Gossip is an effective technique for strengthening informal employee relationships [3,4]. Negative workplace gossip (NWG) is an “*informal and evaluative discussion in an organisation about another member of an organisation who is not present*” [5,6].

*“Good news never goes beyond the gate, while bad news spreads far and wide.”*

According to previous research findings, gossip occurs in all organisations [7]. Employees often overhear, disseminate, or participate in NWG with other employees, who are not part of the conversation, whether they are talking so knowingly or unknowingly [8]. Furthermore, the exploitation of workplace norms, gossip is classified as delinquent political behaviour, or the practice of engaging in social interactions so that it degrades other employees in terms of competition or politics [9]. While most research has looked into the causes of gossip, few have looked into the consequences [5]. Previous studies have shown that NWG may be detrimental to both employees and organisations [5,10]. Those who are the targets of gossip may experience emotional discomfort and stress [10,11,12]. Consequently, gossip is linked to negative behaviours including spreading rumours about others and undermining someone’s efforts [13]. In comparison to other negative behaviours (such as rudeness, sabotaging, and aggressive behaviours), gossip is sufficiently distinguishable from other negative behaviours. Gossip may consist of both implicit and explicit conduct and is considered indirect since it occurs when the intended recipient is not there [11,14]. As a result, gossip is a kind of indirect assault that may put others at a disadvantage while avoiding confrontation with the targets [15,16]. Unlike other forms of direct maltreatment, it is difficult for the intended recipient to pin down the exact nature of the rumours being spread about them as well as to identify both the gossiper and the gossip receiver, making it vulnerable to ambiguity and confusion [3,17,18].

The negative impact of NWG on employees has prompted it to become a hot topic in the field of organisational behaviour [19,20]. Employees see gossip as an essential informal communication technique at work, and they often engage in it to acquire and share information [21,22], as well as to fulfil their social interests [16]. Because of its widespread use, academics have focused greatly on how it affects organisations. [16] found that workplace gossip may be beneficial to Organisations, groups, and gossipers to a degree, but that targeted employees might experience substantial damage mainly [2], because individuals are more sensitive to bad occurrences than good events [23].

The impact of NWG on targets has been studied from a variety of perspectives, including social exchange theory [19], COR [2], self-consistency theory [24], resource-based perspective [9], and social identity theory [25], these paradigms produced insightful findings. Several studies showed the turn down impact of NWG on targets’ attitudes and behaviours, including lower Organisation-based ego [11], lower organisational recognition [9], increased organisational negativity [22,26], stimulating low feelings [27,28] and knowledge hiding [9], and resulting in lower engagement [29], lower creative behaviour [15], low proactive activity [30], and decrease in organisational citizenship behaviour [29].

**Figure 1:**
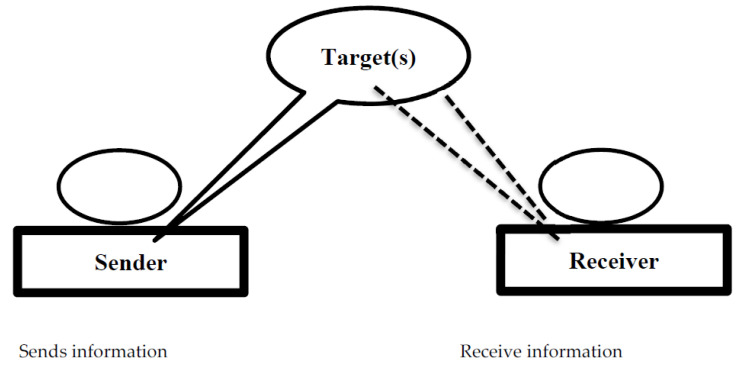
Gossip triad [2,34]. Note: The gossip triangle has three “actors”: sender, target, and receiver.

Although previous research has shown the behavioural consequences of becoming the target of gossip [4,31,32], at the same time little attention was given to the targets’ counterattack to mitigate the pessimistic effects of NWG within organisations. According to the current research, NWG has an emotional impact on employees, and employees may deal with this circumstance by participating in political acts (PA)—“*action designed to attain a purpose by the use of political power or by activity in political channels.”* Our research used affective events theory (AET) [33] to address this gap and decode the emotional repercussions of NWG along with associated behavioural reactions through PA. The idea that humans are passionate and that emotions influence their behaviour underpins AET. We concluded that an essential feature of gossip involves a sender who conveys information about a target to a recipient (see Figure 1). The most often mentioned distinguishing feature was that the gossip target had to be either ignorant of or absent from the conversation.

**Figure 2:**
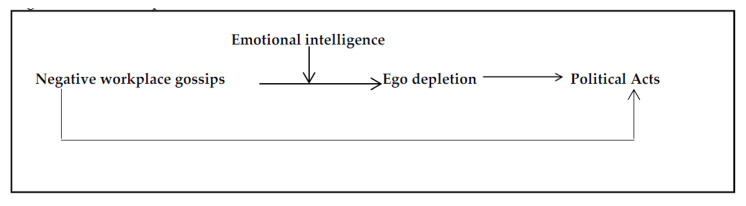
The Conceptual Model.

This research adds to the existing body of knowledge in three ways. First, our study expands the literature on proactive behaviour (political acts) to counter NWG by drawing on AET [33] and using the model of proactivity [2]. It offers a theoretical framework for investigating the factors (i.e., ego depletion) leading to PA. Second, various perspectives are still needed to understand better the underlying mechanism of how NWG affects target behaviour. Prior research in this area has mostly focused on changes in targets’ emotions about their job roles and organisation: unpleasant temperament, organisational-based ego, and organisational commitment [35]. However, NWG may elicit cognitive reactions in employees exposed to it because it is a common, indirect, and unpleasant occurrence of targets [24]. Employees, for example, may not be aware that they are the focus of gossip until they notice their co-employees’ odd conduct and the weird atmosphere in which they are working [36]. According to [37], all these elements of being targeted diminish sentiments and emotions that are associated with attention and cognitive tasks, resulting in a state of ego depletion (ED)—“*ED refers to the phenomenon that self-control performance declines from an initial self-control task to a subsequent one repetition”* [38]. We may obtain a thorough knowledge of the underlying mechanism of ED by studying NWG. Third, from the perspective of AET, emotional intelligence (EI) may help mitigate the direct connection between NWG and ED. The model is shown in Figure 2.

*EI, “the ability to carry out accurate reasoning about emotions and the ability to use emotions and emotional knowledge to enhance thought”* [39].

EI is a state of individual capability that describes an emotional resource that may positively reflect and reduce the connection between NWG and ED [2]. A framework for investigating the significance of EI as a moderator between NWG and ED is proposed in this research based on previous findings and the rationale stated by [2] in their study.

## 2. Theory and Hypothesis Development

### 2.1. Affective Events Theory (AET)

The AET was developed by [40], who also included emotional experience as a supplement to prior cognitive assessment models to explain employee work behaviours and emotions. According to AET, employee emotions and behaviours are influenced by workplace events [40]. Instead, workplace events, such as treatment received from others, interaction characteristics, elicit affective reactions, either positive or negative [33]. When things go well at work, employees feel good about themselves, and when something bad happens at work, it tends to make employees feel down [35]. According to AET, NWG is a negative workplace occurrence since it is a kind of indirect attack or deviant behaviour aimed at the target, and employees’ emotional responses expressed as a negative mood would impede the target’s ego [2,31,41]. As a result, people are frequently involved in political activities to reduce the impact of NWG, which depletes the ego of targets [42]. Consequently, this research establishes a connection between NWG and PA and the subsequent behavioural responses for the following reasons. First and foremost, NWG includes information that tarnishes the performance and capabilities of the targeted in their respective roles, which may aggravate their egos [2,35]. Second, employees are worried about their outward images related to their professional career threatened as a result of NWG, which may lead them to participate in political activity [41]. Third, after being subjected to negative gossip, target employees become protective about their image [43]. Consequently, we propose that participating in political activities may help mitigate the unfavourable impact of NWG on an employee’s ego.

According to AET, individuals actively want to restore their ego by demonstrating EI in the workplace. EI may be considered a distinctive variable of individual resources to back their ego [44]. EI is an individual’s mental capacity to recognise, analyse and utilise emotions, appraise their emotional states. It is an essential resource for employees who need to self-regulate in stressful circumstances [45]. As a result, people with high EI can efficiently restore their self-regulation resources when depleted. On the other hand, self-regulation resources are depleted in those with poor EI.

### 2.2. Negative Workplace Gossip and Political Acts

Acts of politics in the workplace are purely self-serving activities that increase self-interest via influence over other Organisation members’ thinking, perception, or conduct [46,47]. Employees participate in such conduct for their advantage because it allows them to obtain essential resources such as awards, favour, and reputation by behaving politically [48,49]. Employees may utilise informal connections to affect the allocation of Organisational resources and, therefore, engage politically, which may effectively achieve their goals [50]. Additionally, it may have a detrimental impact on the attitudes of other employees of the organisation and may even impact the organization’s overall objectives [23,51]. It is critical to identify the factors that lead to PA. Our research indicates that the targets of NWG may have significant reasons to participate in PA as a retaliatory measure to defend their self-esteem. According to AET, individuals are very sensitive to their emotional state and tend to conserve, guard, and acquire emotions.

Additionally, AET emphasises that people want to spend their time in political activities to heal from and combat emotional losses [40]. As a result, targets are more inclined to behave in self-serving ways to maximize their advantages, such as political activities to solve this unpleasant condition, minimise their losses, and seek more accessible resources. First, NWG often involves unfavourable assessments of the target, endangers their reputation, and may adversely affect their professional status. People who hear it are more inclined to accept it, regardless of whether it is true [52].

Moreover, prestige and professional ability are important workplace resources that help targets compete for Organisational benefits (i.e., perks and privileges) and other career possibilities [32]. Second, the targeted person loses support and cooperation from colleagues due to NWG [26,53]. Third, when targets become aware of negative talk, they become uncomfortable and anxious because they have a negative effect on their reputation and even their professional growth [54]. Targets consume their efforts and attention to identify the source of the rumour and determine how to deal with them—resources that would otherwise have been invested in their job in return for a more meaningful result [55]. Overall, targets are confronted with many detriments and difficulties, and therefore feel compelled to influence the thinking, attitudes, and actions of other organization members to reclaim support and achieve desirable outcomes [2]. They are more likely to participate in political activities to pursue their objectives, such as restoring bonds and resources (e.g., feelings and emotions) inside the organisation and then using them to reverse unfavourable situations. The feelings of confusion generated by the NWG may also contribute to PA [2,11,32].

**Hypothesis** **1** **(H1).** *Negative workplace gossip positively effect political acts.*

### 2.3. Ego Depletion as a Mediator

Affective events theory (AET) describes how feelings and emotions affect the performance of targets in their working life and the other tasks they perform. Employees’ reactions to events that occur at work are described in terms of internal variables, such as personality, emotions, and cognition. In reference [40], the authors believe that affective work behaviours are described by employee mood and emotion, while cognitive-based work behaviours are excellent indicators of employee positivity. Previous findings stated emotions and feelings play a significant role in employees responding to and dealing with difficult circumstances in their places of employment by repressing emotions and exercising self-control over impulsive conduct [56,57]. Individuals slip into a condition of ED if their emotions and moods are exhausted or challenged and cannot be refilled in a healthy manner [33,58], taking risks or participating in immoral activities [59].

NWG tends to induce ED because it tends to devour the feelings and emotions of its targets. This may occur in a variety of ways. First, the targets’ moods and emotions will be disrupted because their time and attention will divert from their normal activities to the wake of examining that they are being targeted [60]. However, they may notice odd behaviours and a weird atmosphere among their co-workers and continue to spend time (e.g., political activities) studying this confusing gossip until they conclude that they are being gossiped about [31,61]. Furthermore, since targets may worry about whether they did anything incorrectly, this thought process may take a long time, using more attention and cognitive resources. Second, when it is verified that they are the subject of rumours, the targets typically feel a depressed state of mind [62]. They may be humiliated by the exposure of personal information, concern about their reputation being harmed, and despairing. All will likely deplete their ego of these negative feelings. Third, according to researchers, targets are more likely to be ignored by their colleagues, making it more difficult for them to carry out their jobs [63]. As a result, individuals must be more careful about how they talk and behave, hastening the depletion of their emotions. We believe that NWG leads targets to lose their feelings and suffer ED because of the above mentioned reasons. NWG victims are more inclined to engage in political activities to maintain their moods and emotions and avoid additional losses [2,64].

**Hypothesis** **2** **(H2).** *Emotion depletion mediates the positive relationship between negative workplace gossip and political acts.*

### 2.4. Emotional Intelligence as a Moderator

Previous studies have shown that the target may be able to detect NWG, and how much of an impact it has depends on the target’s emotional state [11,24,31]. In other words, the extent to which people are aware of and respond to how others treat them has a significant effect on employees’ emotions. The significance of emotions in organisations has generally been overlooked as a topic that is unsuitable for serious study in organisational contexts [65]. Emotion research had minimal effect on the mainstream of organisational research until the late 1980s, despite being a significant resource for employees during stress [40,66,67].

Affective events theory [40] asserts that employees’ experiences in the workplace are characterised by ‘hassles and uplifts,’ which Weiss and Cropanzano refer to as ‘affective events.’ According to AET, positive or negative emotions in employees result from a series of positive or negative affective experiences, which predict attitudinal states and behavioural reactions. For instance, an employee is targeted by NWG (an emotional event), and the target’s ego is affected (an affective state), causing them to suffer in their professional responsibilities (an attitudinal state) [27]. When people participate in self-regulatory activities to a certain degree, they may replenish the continuously depleting self-regulation resources [68]. Individuals benefit from EI by gaining more resources that they can utilise to replace the resources that have been depleted by NWG [69]. According to prior studies, EI, a unique resource characteristic variable, may be considered a crucial psychological resource that can assist people in self-regulating in stressful circumstances. A high EI allows individuals to obtain more role energies by successfully executing different roles, experiencing more pleasurable feelings, and obtaining additional contextual resources via interpersonal interaction [70]. The more EI someone has, the easier it will be for them to replenish their ego’s depleted resources over time. On the other hand, individuals with low EI cannot effectively convert their self-regulatory resources once they have been depleted [71]. Consequently, individuals lack the resources necessary for self-regulation, amplifying the positive effects of work pressure on the ED.

**Hypothesis** **3** **(H3).** *Emotional intelligence moderates the relationship between negative workplace gossip and emotion depletion such that the relationship is weaker for employees with high emotional intelligence than for those with low emotional intelligence.*

### 2.5. Moderation Mediation Examination

Our study so far has supported a theoretical framework in which ED mediates the effect of NWG on PA, and EI works as a moderator that weakens the impact of NWG on ED. Employees with low EI who are subjected to NWG may become politicized. A moderated mediation hypothesis, in which the EI of the targets mitigates the indirect effect of NWG on PA via ED makes sense as a result [2,72]. As previously mentioned, higher degrees of EI, in particular, will weaken the effect of NWG on targets’ ED and ease of the indirect influence of NWG on PA via ED [2]. On the other hand, those with lower levels of EI will see an increase in the impact of NWG on their targets’ ED, as well as an increase in the indirect influence of NWG on PA through the exact mechanism [69,70,71].

**Hypothesis** **4** **(H4).** *The indirect effect of negative workplace gossip on targets’ political acts via emotion depletion is moderated by targets’ emotional intelligence. This relationship is weak when the targets’ emotional intelligence is higher.*

## 3. Method

### 3.1. Sample and Procedures

The data for this research were collected via a three-wave time-lagged study, with a month between each data collection phase, which was completed in three waves. Using this technique, the potential of common method bias was reduced to the absolute minimum [2,73]. Five Pakistani private Organisations, that is, the Saif group of industries (Saif Power limited, Saif textile mills limited, Kohat textile mills limited, Saif health care and Saif energy private limited) were chosen for this study; all the head offices were based in Islamabad. We select these five organizations and also have easy access to these organizations for data collection. We sent questionnaires to employees with the assistance of the Human Resources Department. At the start of the study, each participant was given a number that could be linked to each data collection step. The initial survey (T1) included basic demographic data and assessments of the NWG and EI. Employees were asked to rate ED in the second survey (T2), while the third survey gathered information regarding PA (T3). The employees individually assessed each of these factors.

After distributing questionnaires to 447 employees, we obtained 393 valid responses, resulting in an 87.91% response rate in the first stage. We followed up with the employees who had previously answered the second questionnaire, and 344 valid responses were received, for an overall response rate of 87.53% for the second round of the survey. The final questionnaire a month later, as part of stage three, employees who had replied to the previous stage. During stage three, we received a total of 277 complete and usable questionnaires, resulting in a response rate of 80.5%. A total of 277 employees responded, with 17.32% being female and 82.67% being male. A total of 4.69% had a high school diploma, 12.27% had an HSSC (higher secondary school certificate), 60.28% had a bachelor’s degree, and the remaining 22.74% with a Master’s degree or above. A total of 73.28% of those had between 1–7 years’ of experience. Only 2.16% of employees were over 25 years of age (see Table 1).

### 3.2. Measures

All of the variables were evaluated on a Likert scale from 1 to 5, with 1 being strongly disagree and 5 strongly agree.

#### 3.2.1. Negative Workplace Gossip (α = 0.79)

The three-item scale developed by [10] was used to assess negative workplace gossip. An example of this was “As recently as 1 month ago, false allegations were made about you”.

#### 3.2.2. Ego Depletion (α = 0.84)

We assessed ED in participants by using five questions from the [74,75] measure, which was based on the work of [76]. A sample item was “Generally speaking; I feel like my willpower is gone”.

#### 3.2.3. Political Acts (α = 0.81)

We assessed acts using a four-item scale developed by [77], modified from research conducted by [78]. A sample item was “I spent time winning the approval of those (e.g., supervisors, managers) who can help me”.

#### 3.2.4. Emotional Intelligence (α = 0.93)

We utilised a 16-item version of the Wong and Law EI Scale (WLEIS) to assess EI, which included four dimensions: Self-emotion appraisal (SEA), Others’ emotion appraisal (OEA), use of emotion (UOE), Regulation of emotion (ROE) [79]. A sample item was “I really understand what I feel”.

#### 3.2.5. Control Variables

The previous study’s findings [2] have shown statistically significant connections between demographic variables and PA. The characteristics of our employees’ gender, age, education, and tenure were thus closely regulated. For dummy coding, we used the following codes: male marked as “1” and female marked as “2”; HSSC or less coded as “1,” a bachelor’s degree marked as “2,” and a Master’s degree or above tagged as “3”.

## 4. Results

### 4.1. CFA and Common Method Variance (CMV)

Through maximum likelihood estimation, CFA was performed to evaluate the discriminant validity of all variables using AMOS V. 23. As shown in Table 2, the findings of the CFA indicate a better match (χ2/df = 1.03, RMR = 0.02, GFI = 0.90, CFI = 0.92, RMSEA = 0.03). Compared to the other models, the predicted four-factor model performed better. Additionally, to eliminate the possibility of CMV, which may occur when data are collected from a single source in behavioural research. Harman’s single-factor technique was used in this study. As reported in Table 2, the one-factor model was found to be poorly fitted across all goodness-of-fit criteria (χ2/df = 5.42, RMR = 1.03; RMSEA = 0.14; GFI = 0.44, CFI = 0.34); however, there was a good fit for the four-factor model, indicating that CMV was not a problem in this particular dataset.

### 4.2. Descriptive Statistics

Data for all variables are shown in Table 3, which includes descriptive statistics, reliability coefficients, and correlation coefficients. According to the results of this study, NWG showed a substantial positive connection with ED (r = 0.44, *p* < 0.01) as well as with PA (r = 0.49, *p* < 0.01), and ED was significantly associated with PA (r = 0.43, *p* < 0.01).

### 4.3. Hypothesis Testing

**Figure 3:**
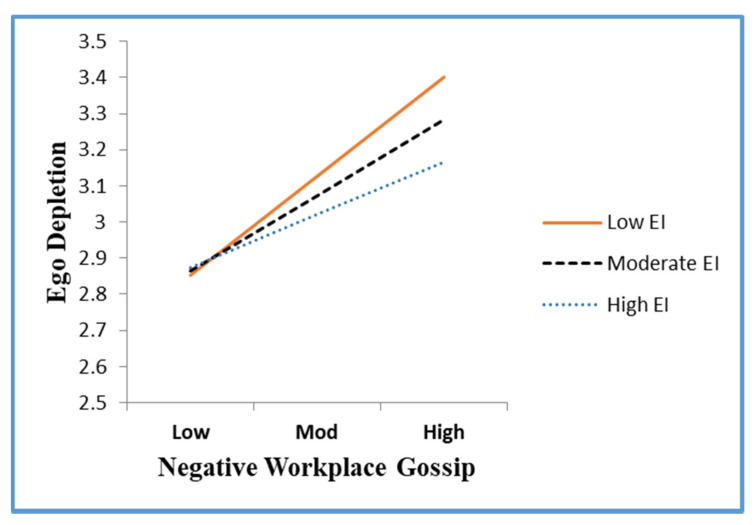
The interactive effects of NWG and EI on ED.

Hierarchical multiple regression analysis was used to examine Hypotheses 1 and 2. Table 4 shows that NWG was positively linked to PA (β = 0.25, *p* < 0.001, Model 5). As a result, hypothesis 1 was confirmed. Hypothesis 2 suggests that ED mediates the link between NWG and PA. Based on the results, (1) a high level of NWG was linked to lower levels of ED (β = 0.44, *p* < 0.001, Model 1), (2) depletion of one’s ego was shown to be positively associated with political behaviour (β = 0.19, *p* < 0.001, Model 6), and (3) the positive effect of NWG on PA decreased after ED was placed (β = 0.13, *p* < 0.001, Model 7). Thus, Hypothesis 2 is validated. Additionally, we determined the statistical significance of the indirect impact using [80] PRODCLIN method. There was no zero in the 95% bias-corrected confidence interval, indicating that the ED caused by NWG had a significant indirect effect on PA β = 0.13, [0.0502, 0.2285]. Thus, Hypothesis 2 received more support. Hayes’ (2013) PROCESS macro (Model 1) was used to determine whether EI could act as a moderator (Hypothesis 3). In accordance with the findings shown in Table 4, it was found that the relationship between NWG and EI was positively linked to ED (β = 0.014 *, *p* < 0.05, Model 8). To make the moderating impact of EI more visible, this research computed two kinds of EI mean: one with a standard deviation and the other with a lower standard deviation, as suggested by [80]. Figure 3 depicts the interactive mode, confirming Hypothesis 3. The moderated mediation was tested using [80] bootstrapping method (Hypothesis 4) as shown in Table 5. EI levels substantially influence the indirect effects of NWG on PA due to ED (Δβ = 0.03). NWG had a greater indirect impact on PA when EI was poor, as shown by the results (β = 0.44 **, *p* < 0.001) than when it was high (β = 0.33 **, n.s.); therefore, Hypothesis 4 is confirmed.

## 5. Theoritical and Practical Implication

### 5.1. Theoretical Implications

There are four distinct ways in which this study adds to the current body of knowledge. First, by emphasising the possibility for self-oriented actions, that is, PA, to evolve as a somewhat proactive reaction aimed at mitigating the adverse consequences of NWG, improves our understanding of negative workplace gossip in the workplace. Although previous research has studied the consequences of targets’ work-related behaviours, it has mainly focused on the influence of targets’ in-role and out-of-role actions in the workplace [81,82]. Moreover, from a cognitive viewpoint, these measures are intended only to preserve feelings by decreasing commitment to work. However, the AET [40] suggests that when employees are confronted with a decrease in emotional well-being, they may take extra activities to maintain positive moods and feelings rather than simply avoiding ED. As a result, prior research has overlooked one crucial kind of self-beneficial workplace behaviour: PA, which may help improve one’s attitude and feelings. Our study sheds light on this by demonstrating how NWG may lead to political action. Second, our results add to the body of knowledge on PA by delving into the frequent but belittled issue of the NWG. Following prior results, only a small number of forerunners of PA have been confirmed in earlier research. They have focused mainly on the impact of individual and situational variables, such as the accomplishment of employees’ goals and the organization’s political environment, leaving a broad variety of working circumstances untouched in their investigations. Researchers have previously urged greater attention to this subject to better understand why employees engage politically in their jobs [83,84,85]. As a result, by adding NWG as a motivator of PA, the current research contributes to the existing literature in a valuable way. Third, this research sheds insight into the underlying process of ED, which is responsible for the detrimental effect of NWG on PA. In light of AET, we point out that NWG results in a significant loss of emotions, which leads to ED and further undesirable political behaviour on the part of employees [2]. According to our results, NWG is a resource-draining event that produces ED, which encourages PA. We emphasise the importance of ED while also providing insights into the PA that may emerge as a consequence of ED. Fourth, by examining at the moderating effect of EI, this study shows an essential boundary condition for the impact of NWG. NWG is toxic to targets with have low EI because they are more likely to investigate the possibility of being gossiped. As a result, they experience a higher level of ED, leading to engaging in PA. We extend the current research on the degree to which people may defend self-interested behaviour affected by NWG by demonstrating EI. Furthermore, we add to AET by giving empirical evidence that a person’s resource process is affected by their emotions and feelings.

### 5.2. Managerial Implications

Our findings emphasise the need for strong management and organisational reaction to NWG, which affects both individuals and organisations in. NWG has previously been shown to be a source of stress [2,24]. According to previous studies, targets’ job performance and workplace behaviour are negatively affected by NWG, which is a significant negative factor in the working environment [26,30]. We classify the managerial contributions of our research into three major categories based on the results of our empirical investigation. First, managers should consider taking practical measures to avoid and control NWG to minimise its occurrence in an organisation and prevent victims from being victimised. Managers may establish value-based standards or procedures that make it evident that NWG is not allowed in the workplace (for example, prohibiting talking about other employee’s privacy), backed up by penalties for those who consistently break the standards. Creating organisation-wide procedures to support this zero-tolerance approach will guarantee that individual incidents of NWG are dealt with early to avoid spreading and offer appropriate methods for employees to notify management of unfavourable circumstances or individual targeting. By sharing accurate information promptly regarding individual performance, bonuses, pay and privileges, reforms, and inconsistencies, organisations may minimise the likelihood of NWG. Regular team interactions, one-to-many talks, and routine activities such as team picnics may help reduce NWG [86,87]. Second, managers should focus on avoiding employee ED. Our findings indicate that those who focus on NWG are more prone to suffer from ED and engage in political activities. Our findings emphasise the significance of preventing targets from experiencing ED at work, as this would enhance their EI rather than induce self-serving actions.

Consequently, managers should be alert for indications of ED in their employees via frequent communication and monitoring of their actions. They should intervene as soon as possible to assist them in replenishing their resources and recovering from the ED [7]. To increase targets’ self-esteem, managers may provide encouragement and advice to help them improve their work-related concentration. They could propose flexible working hours to allow them to acquire cognitive and emotional resources. Organisations may also assist employees in improving their emotional stability and coping strategies when faced with gossip by providing suitable training and organising team-building activities. When ED is already an issue, this may be done proactively rather than reactively.

Third, we conclude that our findings also suggest that the higher an employee’s degree of EI the more insignificant an impact NWG has on ED. Managers might consider adjusting their recruitment and training techniques to reflect this. Our findings indicate that Organisations would be wise to choose employees with high emotional stability and strength levels to minimise the effect of NWG on ED on their staff. While at the same time, managers should offer employees training to improve their EI to minimise ED and the likelihood of their being engaged in political activities.

## 6. Limitation and Future Avenues

The first issue related to this research is that it relies on self-reporting by the participants, which is problematic since NWG, ED, EI, and PA are all subjective emotions. This measurement technique has the potential to cause CMB [88,89]. Although the statistical analyses revealed no evidence of a CMB issue, the objective measurement technique may be more informative. Second, it is not easy to accurately determine a causal connection when utilising time-lag data. For the findings to be more compelling, longitudinal studies should be conducted to establish the causal relationship between the factors involved. Third, other psychological factors, such as neuroticism and self-consciousness, may play a role as mediators in the process by which NWG leads to PA, further reducing people’s natural capacity to execute at work. Fourthly, the connection between NWG and PA may be investigated from a variety of theoretical perspectives, including self-verification theory, in addition to AET. Finally, for practical purposes, future studies should analyse coping strategies to reduce the impact of NWG on employees’ ED, such as trait mindfulness and forgiveness, self-monitoring, and other self-regulation methods.

## 7. Conclusions

**Figure 4:**
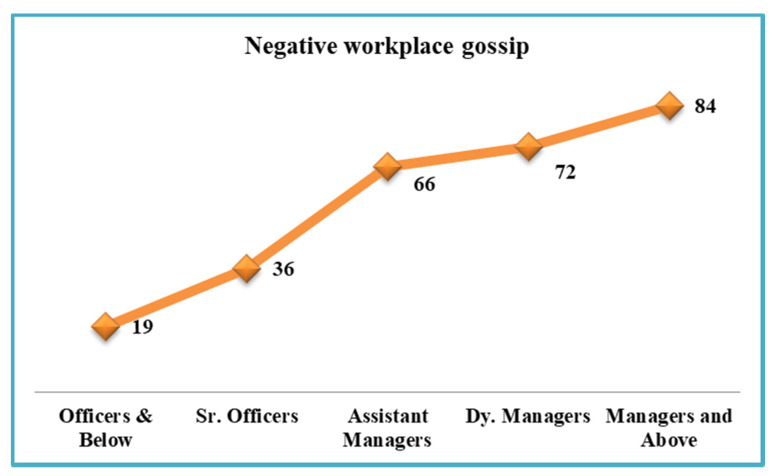
Hierarchy wise NWG comparison.

Almost all employees will be the target of negative workplace gossip at some time in their careers, leading them to suffer unfavourable consequences and pushing them to seek methods to rectify the situation before it becomes irreversible. Using AET, this study aims to show how NWG may drain one’s ego while simultaneously motivating one to participate in PA. Furthermore, by examining the moderating effect of EI, we can better understand the process’s model parameters. Line managers must be aware of NWG, minimise its incidence, and reduce its negative effects. This study contributes to the existing body of knowledge and stimulates new ways of thinking about the subject. To make the study design even better, we also circulated a 1 item questionnaire open-ended “Repeated a rumour or gossip about colleagues or manager at work” to express their experiences about their colleagues and immediate bosses to know hierarchy-wise gossip positions. We found that managers (e.g., immediate bosses/line managers) were more involved in NWG than others 84 (30%) out of 277 (see Figure 4). Many employees were under the impression that my boss/line manager called their offices weekly for intense discussions about co-workers or other departments. I have heard my boss say some nasty comments about their colleagues and then attempt to drag me into the conversation, (“What do you think about X? Do you think W/Y/Z is incompetent/hysterical?”). They may rehash previous dramas/gossip and express their delight that W/Y/Z is no longer in the picture. At present, we have little option but to stay neutral, shrugging our shoulders or stating “we don’t know” when someone attempts to manipulate to say anything negative about someone.

Managers and above are the most likely source of Gossip initiation. Their primary focus is to sustain their position and to keep themselves aware of their surroundings. Gossips work to assess the situation and develop guidelines for managing the departments and maintaining their jobs. We conclude, based on our results, that bosses should be nice and polite and interested in their employees’ well-being, but the kind of colleague with whom you gossip should be someone outside the workplace (though you should not be talking about someone in reality). When you breach that threshold, you not only create an uncomfortable atmosphere, but you may also create ED among employees, causing them to lose interest in their jobs and engage in political acts to counter.

## Figures and Tables

**Table 1:** Sample Characteristics.

Demographic Variables	Frequency	Percentage (%)
Gender		
Male	229	82.67
Female	48	17.32
Age (yrs)		
21–30	43	15.52
31–40	103	37.18
41–50	98	35.37
51–60	33	11.91
Tenure		
1–7 years	203	73.28
8–15 years	61	22.02
16–25 years	07	2.52
Over 25 years	06	2.16
Qualifications		
High school	13	4.69
HSSC	34	12.27
Bachelor’s degree	167	60.28
Master’s degree or above	63	22.7

**Table 2:** Results of the confirmatory factor analysis.

Model	χ2/df	RMR	GFI	CFI	RMSEA
Baseline model (four-factor model)	1.03	0.02	0.90	0.92	0.03
4-factor model ^a^	4.39	0.08	0.93	0.94	0.09
3-factor model ^b^	2.54	0.04	0.94	0.93	0.07
2-factor model ^c^	4.69	0.05	0.97	0.96	0.05
1-factor model ^d^	5.42	1.03	0.44	0.34	0.15

^a^ Combining NWG and ED; ^b^ Combining NWG, ED, and PA; ^c^ Combining NWG, ED, and EI; ^d^ Combining all items.

**Table 3:** Mean, standard deviation, reliability and correlations of this study (*n* = 277).

Variables	Mean	S.D	CR	AVE	1	2	3	4	5	6	7	8
1. Age			2.44	0.84	−0.001							
2. Gender			1.38	0.49	−0.024							
3. Education			2.78	0.54	−0.004	−0.199 **	0.056					
4. Tenure			1.99	0.68	0.189 **	−0.039	−0.042	0.071	(0.81)			
5. Negative workplace gossip	3.47	0.50	0.89	0.53	0.097	0.120 *	0.112	−0.066	0.441 **	(0.83)		
6. ED	3.10	0.47	0.91	0.56	0.079	−0.055	−0.122 *	0.029	0.490 **	0.437 **	(0.77)	
7. PA	3.12	0.54	0.93	0.53	−0.010	0.051	0.004	0.060	0.035	−0.082	−0.119 *	(0.84)
8. EI	1.52	0.35	0.88	0.51	−0.001							

** Correlation is significant at the 0.01 level (2-tailed). * Correlation is significant at the 0.05 level (2-tailed).

**Table 4:** Results of hierarchical regression analyses (*n* = 277).

	Ego Depletion				Political Acts			
	M1	M2	M3	M4	M5	M6	M7	M8
Control Variables								
Age	−0.009	−0.007	0.008	0.054	0.051	0.024	0.047	−0.004
Gender	−0.040	0.037	0.131	0.119	0.119	−0.121	0.113	−0.023
Education	−0.102	0.004	0.114	0.098	−0.122	−0.174	0.099	−0.106
Tenure	−0.005	0.030	−0.068	−0.043	0.029	0.046	−0.057	−0.003
Independent Variable								
NWG	0.44 ***				0.24 ***	0.13 ***		
Mediator							0.19 ***	
ED								
Moderator								
EI		0.007						
Interaction								
Negativeworkplacegossip								0.014 *
x EI								
F	2.59	33.08 ***	31.83 ***	27.08 ***	31.05 ***	31.63 ***	33.72 ***	31.07 ***
ΔF	2.60	62.98 ***	23.82 ***	1.63	4.82	4.66	4.66	4.89
R^2^	0.009	0.195	0.25	0.28	0.31	0.31	0.33	0.31
ΔR^2^	0.006	0.189	0.23	0.004	0.012	0.011	0.011	0.014

Note(s): *n* = 277; *** Correlation is significant at the 0.001 level (2-tailed) and * Correlation is significant at the 0.05 level (2-tailed).

**Table 5:** Results of moderation mediation (*n* = 277).

Moderator Variable	NWG (X) → ED (M) → PA (Y)				
	Stage		Effect		
	First	Second	Direct Effect	Indirect effect	Total Effect
	P^MX^	P^YM^	P^YX^	(P^YM^ P^MX^)	(P^YX^ + P^YM^ P^MX^)
Simple paths for low EI	0.41 **	0.13 **	0.39 **	0.05	0.44 **
Simple paths for high EI	0.28 **	0.09	0.31 **	0.02	0.33 **
Differences	0.13 **	0.04	0.08	0.03	0.09
	Index of moderated mediation				
	Index	BootSE	BootLLCI	BootULCI	
EI	−0.1142	0.0557	−0.2380	−0.0212	

P^MX^: NWG → ED; P^YM^: ED → PA; P^YX^: NWG → PA. One standard deviation below the mean value of ED is referred to below EI. High EI refers to one standard deviation above the mean value of ED. Bias-corrected confidence intervals derived from bootstrapping estimations were used for direct, indirect, and total effects tests. ** *p* < 0.01.

## Data Availability

The data presented in this study are available on request. The data are not publicly available due to privacy reasons.
